# Chromosome screening using culture medium of embryos fertilised in vitro: a pilot clinical study

**DOI:** 10.1186/s12967-019-1827-1

**Published:** 2019-03-08

**Authors:** Rui Fang, Weimin Yang, Xin Zhao, Fang Xiong, Caiqing Guo, Jianping Xiao, Li Chen, Xiaoqing Song, Honghua Wang, Jie Chen, Xiao Xiao, Bing Yao, Li-Yi Cai

**Affiliations:** 1 Centre for Reproductive Medicine, Wuxi Maternity and Child Health Hospital Affiliated to Nanjing Medical University, Wuxi, 214002 Jiangsu China; 2 Wuxi Maternity and Child Health Hospital Affiliated to Nanjing Medical University, Wuxi, 214002 Jiangsu China; 3 Centre for Reproductive Medicine, Hebei Maternity and Reproductive Hospital, Shijiazhuang, 050090 Hebei China; 4 Reproductive Medical Center of Nanjing Jinling Hospital and the Collaborative Innovation Platform for Reproductive Biology and Technology, Nanjing University School of Medicine, Nanjing, 210002 Jiangsu China

**Keywords:** Non-invasive chromosome screening, Assisted reproductive technology, Chromosomal ploidy, Next-generation sequencing, Clinical outcome

## Abstract

**Background:**

Previous studies from this as well as other research groups suggested that non-invasive chromosome screening (NICS) with embryo culture medium can be used to identify chromosomal ploidy and chromosomal abnormalities. We here report a series of clinical cases utilizing the technology.

**Methods:**

A total of 45 couples underwent in vitro fertilisation during a period between February 2016 and February 2017. Karyotyping revealed normal chromosomes in both partners in 23 couples, and chromosomal rearrangements in at least one partner in 22 couples. Intracytoplasmic sperm injection (ICSI) was used for fertilization. NICS was carried out using embryo culture medium at the blastocyst stage via multiple annealing and looping-based amplification cycles, whole-genome amplification and next-generation sequencing.

**Results:**

A total of 413 embryos were obtained; 170 blastocysts were subjected to NICS. The screening showed euploidy in 79 embryos, aneuploidy in 52 embryos, and mosaic ploidy for 33 embryos. The rate of euploidy was comparable in couples with normal karyotype (50.7%; 38/75) vs. chromosomal rearrangement (43.2%; 41/95). A total of 52 euploid embryos (50 oocyte retrieval cycles) were transferred in 43 women. Biochemical pregnancy rate was 72.0% (36/50). Clinical pregnancy rate was 58.0% (29/50). The rate of spontaneous miscarriage was 3/29 (none with chromosomal aneuploidy). A total of 27 healthy babies were delivered.

**Conclusions:**

NICS could identify embryo chromosomal abnormalities in couples either with or without chromosomal rearrangement, with satisfying clinical outcomes.

**Electronic supplementary material:**

The online version of this article (10.1186/s12967-019-1827-1) contains supplementary material, which is available to authorized users.

## Background

One of the greatest challenges for in vitro fertilisation and intracytoplasmic sperm injection (ICSI) is accurately selecting viable embryos that are more likely to achieve healthy livebirths following implantation. Currently, this selection is based on morphological assessment [1], but embryo morphology does not always correlate with chromosome status. In fact, a substantial proportion of human blastocysts designated as high grade based on morphology has chromosomal aneuploidy [2].

Embryo biopsy and preimplantation genetic screening (PGS) provide more direct assessment of chromosome status, and could improve the rate of implantation and clinical pregnancy [3, 4]. The biopsy procedure involves removing either a single cell at the cleavage stage or several trophectoderm cells at the blastocyst stage [5]. PGS has seen limited clinical application because of technical difficulties and concerns over the long-term health of the offsprings. Animal studies suggest embryo biopsy could delay blastocoel formation and increase the risk of neurodegeneration and dysfunction in the offsprings [6–8].

A less invasive alternative is to analyse genomic DNA from embryo culture medium or blastocyst cavity fluid [9–14]. Culture medium may be a more reliable source because screening of blastocyst cavity fluid often gives results inconsistent with those from embryo biopsy [11–17]. In addition, collecting blastocyst cavity fluid can be as challenging as embryo biopsy.

Previous studies from this research group as well as others have shown that DNA testing using embryo culture medium on days 5 or 6 could detect chromosome aneuploidy with resonable positive predictive value and high negative predictive value [18, 19], suggesting that the NICS assay could be used for selecting chromosomally normal embryos.

Here, we report the clinical outcomes of using NICS to select embryos for implantation in a total of 45 couples (22 with normal karyotype and 23 with chromosomal rearrangement in at least one partner).

## Methods

### Study design and patients

**Fig. 1:**
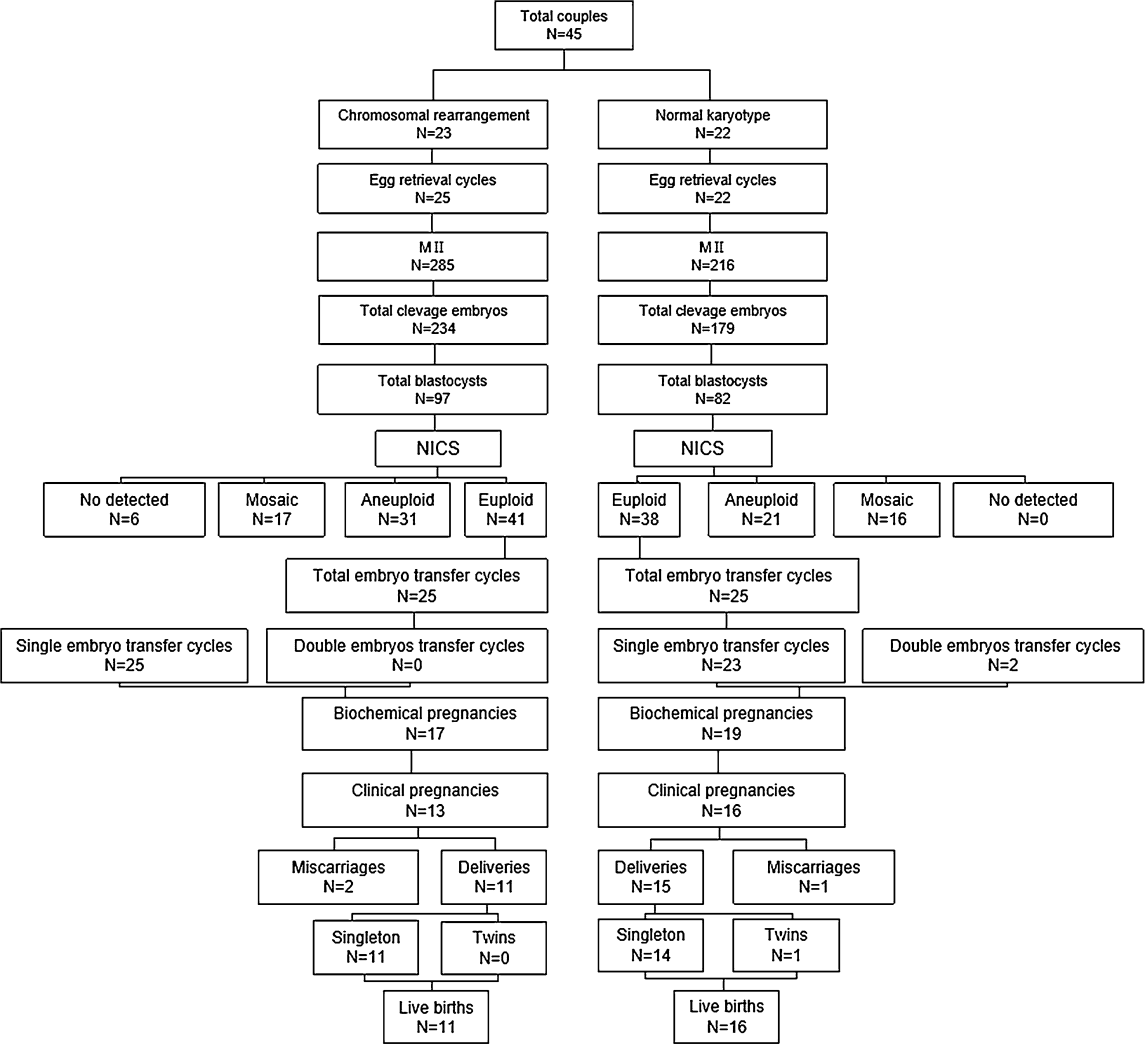
Schematic representation of the study design and work flow

This study was conducted from February 1, 2016 to January 31, 2017, with the approval by the Ethics Committee of the Wuxi Maternal and Child Health Care Hospital. All women had a history of recurrent spontaneous abortion (≥ 3 events) or repeated implantation failure ( ≥ 3 events). All couples had a consultation with a clinical geneticist and were karyotyped. Study design is shown in Fig. 1.

### Ovarian stimulation and oocyte retrieval

Ovarian stimulation was carried out using clomiphene citrate and gonadotropin. Briefly, clomiphene citrate (50 mg/day) was administered orally on an extended regimen from cycle day 3 until the day before induction of final oocyte maturation. Human menopausal gonadotropin or recombinant follicular stimulating hormone was given by injection (150–225 IU/day) from cycle day 4. Ultrasound images and hormone profile (oestradiol [E_2_], luteinising hormone, progesterone) were monitored daily starting on day 8 and until the triggering day. Oocytes were retrieved at 36 h after trigger administration of human chorionic gonadotropin.

### Blastocyst culture

All embryos were fertilised using ICSI. Embryos with two pronuclei were transferred to individual 30-µL droplets of cleavage-stage SAGE culture medium (CooperSurgical Fertility, Malov, Denmark) in a 30-mm Falcon culture dish overlaid with 2.5-mL mineral oil (CooperSurgical Fertility) and cultured at 37 °C in an atmosphere containing 5% O_2_ and 5.5% CO_2_. On day 3 after fertilisation, the embryo was repeatedly pipetted using 135-μm stripper tips (CooperSurgical Fertility), then individual embryos were placed in 30-µL droplets of Quinn's Advantage Protein Plus blastocyst culture medium (CooperSurgical Fertility) and cultured for 2–3 days to the blastocyst stage in an atmosphere containing 5% O_2_ and 5.5% CO_2_ at 37 °C. On day 5 or 6, blastocyst development and quality were evaluated as described [20].

### Sample collection and blastocyst vitrification

Blastocyst culture medium (20–25 µL) was transferred to DNase- and RNase-free PCR tubes containing 5-µL cell lysis buffer (Yikon Genomics, China), snap-frozen in liquid nitrogen, and stored at − 80 °C until NICS. Blastocysts were frozen via vitrification and stored in liquid nitrogen (Cryotop Safety Kit; Kitazato Corp., Tokyo, Japan).

### Whole-genome amplification and DNA sequencing

NICS was performed using culture medium as previously described [19]. DNA for whole-genome amplification was amplified using multiple annealing and looping-based amplification cycles (cat no. YK001B, Yikon Genomics). Amplification products were sequenced on an Illumina HiSeq 2500 platform (Illumina, San Diego, CA, USA) with approximately two million sequencing reads per sample. The read numbers were counted along the whole genome with a bin size of 1 Mb and normalised based on GC content and a reference dataset. The number of read counts served as the index of ploidy: a 50% increase indicates an increase in the number of chromosomes from 2 to 3, whereas a 50% decrease indicates a reduction in the number of chromosomes from 2 to 1 [21, 22].

### Endometrial preparation and blastocyst transfer

The endometrium was prepared for transfer of frozen–thawed blastocysts using an artificial cycle [23–25]. Hormone replacement therapy (oral E2 valerate at 6 mg/day) was started on day 3 of the menstrual cycle. Ultrasound was performed, and the serum progesterone level was measured after 12 days of E2 replacement. When the endometrium was 8-mm thick, luteal support was started with daily oral progesterone or vaginal micronised progesterone gel administration (Crinone 8%, Merck Serono KGaA, Darmstadt, Germany). E2 valerate and progesterone administration continued until 10 weeks of gestation.

Blastocysts were selected based on traditional morphological assessment and NICS results, and transferred on day 6 of progesterone administration. Vitrified blastocysts were warmed using Kitazato vitrification thawing solution as described [19]. Before transfer, the cryotop strip of frozen embryos was immersed in thawing solution (1-mol/L sucrose) for 60 s at 37 °C, then in dilution solution (0.5-mol/L sucrose) for 3 min. Blastocysts were washed in washing solution without sucrose for 3–5 min. Surviving blastocysts were incubated in an atmosphere of 5% O_2_ and 5.5% CO_2_ at 37 °C for 1–2 h before transfer to the uterus.

### Clinical outcomes

Biochemical pregnancy was defined as human chorionic gonadotropin  > 10 mIU/mL at 10 days after blastocyst transfer. The rate of clinical pregnancy was calculated as the number of transfer cycles in which transferred blastocysts developed to a stage at which foetal heartbeat was visible by ultrasound, divided by the total number of freeze-thaw blastocyst transfer cycles. The miscarriage rate was defined as the number of spontaneous pregnancies lost, divided by the number of freeze-thaw blastocyst transfer cycles leading to clinical pregnancy.

### Statistical analysis

Continuous variables are reported as mean ± standard deviation. Categorical variables are presented as frequencies. Inter-group differences were assessed for significance using Fisher's exact test. *P* < 0.05 (2-sided) was considered statistically significant. All statistical analyses were performed using SPSS 20.0 (IBM, Armonk, NY, USA).

## Results

### Patient characteristics

A total of 45 couples were included. The average age of the women was 30.7  5.0 years (range 23–42 years). A total of 501 oocytes (47 cycles) in metaphase of the second meiotic division were obtained (Table 1). ICSI produced 421 zygotes and 413 embryos, of which 179 (43.3%) developed into transplantable blastocysts. Karyotyping revealed chromosome rearrangements in 23 couples, including Robertson translocation (n = 4), balanced translocation (n = 10), chromosome inversion (n = 4), and 47XYY (n = 1) (Additional file 1: Table S1). The remaining 4 couples had unbalanced chromosome rearrangements involving deletions or duplications.

**Table 1:** Clinical characteristics of patients for whom NICS was performed on embryo culture medium

	Chromosomal rearrangement (n = 23)	Normal karyotype (n = 22)	Entire cohort (n = 45)
Female age (years)	29.4 ± 5.4	31.7 ± 4.5	30.7 ± 5.0
Female body mass index (kg/m^2^)	21.5 ± 3.1	23.0 ± 3.2	22.3 ± 3.2
Baseline FSH (mIU/mL)	6.8 ± 2.5	6.8 ± 1.4	6.8 ± 2.1
Baseline LH (mIU/mL)	5.0 ± 3.4	5.6 ± 2.4	5.4 ± 2.9
Infertility duration (years)	3.0 ± 1.9	3.2 ± 2.7	3.1 ± 2.4
Number of cycles	25	22	47
CCOCs	345	292	637
MII	285	216	501
Zygotes	237 (83.2%)	184 (85.2%)	421 (84.0%)
Embryos obtained	234 (98.7%)	179 (97.3%)	413 (98.1%)
Blastocysts obtained	97 (41.5%)	82 (45.8%)	179 (43.3%)
Blastocysts subjected to NICS	95	75	170
Euploid	41 (43.2%)	38 (50.7%)	79 (46.5%)
Aneuploid	31 (32.6%)	21 (28.0%)	52 (30.6%)
Mosaic	17 (17.9%)	16 (21.3%)	33 (19.4%)
Unsuitable for implantation by NICS	6 (6.3%)	0	6 (3.5%)
Cycles without transferable blastocysts	4% (1/25)	4.5% (1/22)	4.3% (2/47)

Values shown are n, n (%) or mean ± SD

CCOCs, cumulus oophorus-oocyte complex; FSH, follicle-stimulating hormone; LH, luteinizing hormone; MII, mature oocyte; NICS, non-invasive chromosome screening

### Correlation of embryo morphology with euploidy

Whole-genome amplification and next-generation sequencing of DNA in culture medium from 164 blastocysts revealed that 79 (46.5%) were euploid; 52 (30.6%) aneuploid; and 33 (19.4%) mosaic (Table 2). No usable signal was obtained in the remaining 6 blastocysts.

**Table 2:** Embryo morphology and ploidy

	Euploid	Aneuploid	Mosaic	No result	OR (95% CI)	P
Day 5 embryos	75 (46.6%)	47 (29.2%)	33 (20.5%)	6 (3.7%)	1.09 (0.282–4.208)	0.900
Day 6 embryos	4 (44.4%)	5 (55.6%)	0	0	1	
Morphology						
Expansion						
4	72 (46.5%)	46 (29.7%)	32 (20.6%)	5 (3.2%)	0.867	0.864
5	4 (44.4%)	3 (33.3%)	1 (11.1%)	1 (11.1%)	0.800	0.833
6	3 (50.0%)	3 (50.0%)	0	0	1	
Inner cell mass						
A	35 (49.3%)	23 (32.4%)	11 (15.5%)	2 (2.8%)	0.972 (0.130–7.288)	0.978
B	42 (44.2%)	27 (28.4%)	22 (23.2%)	4 (4.2%)	0.792 (0.107–5.863)	0.792
C	2 (50.0%)	2 (50.0%)	0	0	1	
Trophectoderm						
A	37 (54.4%)	23 (33.8%)	7 (10.3%)	1 (1.5%)	1.432 (0.702–2.924)	0.324
B	17 (36.2%)	15 (31.9%)	11 (23.4%)	4 (8.5%)	0.68 (0.306–1.509)	0.343
C	25 (45.5%)	14 (25.5%)	15 (27.3%)	1 (1.8%)	1	
Embryo morphology grade					E	
1 (AA)	33 (55.0%)	20 (33.3%)	6 (10.0%)	1 (1.7%)	1.504 (0.728–3.108)	0.27
2 (AB, BA, BB)	19 (37.3%)	16 (31.4%)	12 (23.5%)	4 (7.8%)	0.769 (0.359–1.647)	0.50
3 (AC, BC, CA, CB)	27 (45.8%)	16 (27.1%)	15 (25.4%)	1 (1.7%)	1	

Values shown are n (%), unless otherwise noted

Of the 60 embryos assigned morphology grade 1 (AA), 33 (55%) were euploid; of the 51 embryos with morphology grade 2 (AB, BA and BB), 19 (37.3%) were euploid; and of the 59 embryos with grade 3 (AC, CA, BC and CB), 27 (45.5%) were euploid (Table 2). Logistic regression showed no significant association between euploidy and morphology grade. In comparison to the embryos with morphology grade 3, there are no significant difference in the probability of euploidy in blastocysts with morphology grade 1 (OR = 1.504, *P* = 0.27) and 2 (OR = 0.769, *P* = 0.50) (Table 2).

### NICS

Based on NICS of 95 embryos from couples with chromosomal rearrangements, 41 embryos (43.2%) were euploid, 31 (32.6%) were aneuploid and 17 (17.9%) showed mosaic ploidy (Table 1). Ploidy could not be determined in 6 embryos (6.3%).

Based on NICS of 75 embryos from couples with normal karyotypes, 38 (50.7%) were euploid, 21 (28.0%) aneuploid and 16 (21.3%) mosaic. The two groups of couples did not differ significantly in the rate of ploidy abnormalities. Transferable blastocysts were not obtained from 1 oocyte retrieval cycle in each group.

### Clinical outcomes

Twenty-five euploid blastocysts were transferred into 22 patients who underwent preimplantation genetic diagnosis (PGD); three women underwent two embryo transfers. 27 euploid blastocysts were implanted in 21 patients who underwent PGS; the implantation was performed within 25 cycles, with four women undergoing two transplantations and two women receiving two embryos per cycle (Table 3).

**Table 3:** Pregnancy outcomes with NICS

	Chromosomal rearrangement	Normal karyotype	Total
Total ET			
Cycles	25	25	50
Patients	22	21	43
(SET/DET)	25/0	23/2	48/2
Transferred euploid blastocysts	25	27	52
Biochemical pregnancies	68% (17/25)	76% (19/25)	72.0% (36/50)
Clinical pregnancies	52% (13/25)	64% (16/25)	58.0% (29/50)
Miscarriages	15.4% (2/13)	6.2% (1/16)	10.3% (3/29)
Deliveries	11	15	26
Singleton/twins	11/0	14/1	25/1
Babies born (male/female)	11 (6/5)	16 (9/7)	27 (15/12)
Birth weight (g, mean ± SD)	3283.7 ± 412.4	3174.7 ± 391.5	3217.5 ± 403.4

DET, double embryos transfer; ET, embryo transfer; NICS, non-invasive chromosome screening; SET, single embryo transfer

Biochemical pregnancy rate was 68% (17/25) in the PGD group and 76% (19/25) in the PGS group (Table 3). The clinical pregnancy rate was 52% (13/25) and 63% (16/25), respectively. Neither biochemical or clinical pregnancy rate differ significantly between the groups. Miscarriages occurred in 2 of the 13 (15.4%) pregnancies in the PGD group and in 1 of the 16 (6.2%) pregnancies in the PGS group. All three miscarried foetuses were euploid based on foetal tissue examination.

26 women in each group had given birth to healthy babies. Birth weight did not differ significantly between the two groups.

## Discussion

The results from this pilot study suggest that NICS can be used to screen chromosomal ploidy in embryos and identify chromosomal rearrangements. The overall clinical pregnancy rate was 58% (64% in couples with normal karyotype, and 52% in couples with chromosomal rearrangements). The clinical pregnancy rate of 52% in couples with chromosomal rearrangements in the current study is similar to the rate of 45.1% reported in a study of patients with chromosomal rearrangements who underwent PGD based on next-generation sequencing [26].

The clinical pregnancy rate in the current study is apparently higher than that in the ESHRE study, which reported clinical pregnancy rate of 28–30% among patients undergoing PGS and 25–35% among patients undergoing PGD based on PCR, microarray analysis, or fluorescence in situ hybridization [27]. The miscarriage rate in our study (6.2% in the PGS group and 15.4% in the PGD group) is lower than that reported by in the ESHRE study (9% and 18%, respectively). These results indicated that using NICS to detect chromosomal rearrangements and to screen chromosome ploidy is a viable approach to select embryos with high developmental potential.

Consistent with previous studies [28, 29], we did not observe significant correlation between morphological grade and embryo ploidy. These results argue strongly against morphology-based assessment of an embryo's implantation potential. At the minimum, a combination of morphological assessment with NICS-based PGS can further improve in vitro fertilisation outcomes. This should be examined in future work, preferably in prospective randomised studies.

The sex ratio of the 27 newborns in our study was consistent with NICS prediction. 11 newborns in the PGD group underwent amniocentesis prior to the eventual delivery. Similar to PGS involving embryo biopsy, NICS cannot distinguish normal embryos from those carrying a balanced translocation. However, NICS can accurately identify embryos with balanced chromosomes, since observed variations in chromosome copy number were consistent with NICS predictions for the newborns and aborted foetuses (data not shown).

In our previous study, we validated the NICS assay for identification of chromosomal abnormalities by using donated complete embryo and obtained 0.882 sensitivity and 0.840 specificity [19]. The PPV and NPV were 78.9% and 91.3%, respectively. The relatively low PPV indicated significant false positivity, and could reflect the self-repair process in which abnormal DNA fragments are released by early embryos into culture medium during development [30, 31]. Since the goal of NICS assay is to select healthy embryos for implantation, we believe that the assay is clinically useful considering the relatively high NPV.

## Conclusions

Here we demonstrate the usefulness of genomic DNA testing in embryo culture medium, as suggested from previous work [10]. Blastocyst fluid is another source of embryo DNA, but smaller amount of DNA, and thus low rate of detection (63%–76.5%) remains a major challenge. Also, testing results using blastocyst fluid may be inaccurate for preimplantation genetic testing [12–17].

Consistent with previous work using the same method [32], the success rate of amplification with NICS was 96.4% in the current study. This likely reflects the appreciable amount of embryonic DNA in the culture medium volume, which is 100-fold greater than the volume of blastocyst fluid with a similar DNA concentration [10]. In addition, multiple annealing and looping-based amplification cycling may reduce the effects of amplification inhibitors present in the embryo culture. We used culture medium from the blastula stage rather than oocyte stage, which may result in higher DNA amounts because the cell mass is greater and because mosaicism occurs more often at the cleavage stage.

Despite the advantages of using culture medium for NICS, it is vulnerable to contamination with sperm and cumulus granulosa cells. Also, serum in the medium could inhibit DNA amplification. At our centre, embryos are repeatedly rinsed when changing the culture medium on day 3 in order to enhance the removal of cumulus cells and other sources of DNA contamination. Future studies are needed to examine the potential effects of these procedures on embryonic development.

Our results should be interpreted with caution given the retrospective, observational study design and the small sample size. The study is subject to a variety of biases, including but not limited to patient selection. In addition, we did not include a control group in which only morphology scoring was used to screen embryos for implantation. Larger, randomised controlled trials are needed to verify and extend our findings.

## Additional file

**Additional file 1: Table S1.** Karyotypes of the patients with chromosomal abnormalities in the chromosomal rearrangement group.

## Abbreviations

NICS: non-invasive chromosome screening
ICSI: intracytoplasmic sperm injection
PGS: preimplantation genetic screening
PGD: preimplantation genetic diagnosis
