# Supplementary material for: Chromosome screening using culture medium of embryos fertilised in vitro: a pilot clinical study
Source: J Transl Med. 2019 Mar 8;17:73. doi: 10.1186/s12967-019-1827-1 (PMC6408780; doi:10.1186/s12967-019-1827-1)
Supplement: Supplementary file 1 — Additional file 1: Table S1. Karyotypes of the patients with chromosomal abnormalities in the chromosomal rearrangement group. [file 12967_2019_1827_MOESM1_ESM.docx]

**Table S1.** Karyotypes of the patients with chromosomal abnormalities in the chromosomal rearrangement group

| Number | PGD indication |
| --- | --- |
|  |  |
| 1 | 46,XY, t(12:15) (p12:q13) |
| 2 | 46,XY, t(3;11)(q13.3;p14) |
| 3 | 46,XY, t(1;2)(q21;q11.2) |
| 4 | 46,XY, Yqter- |
| 5 | 46,XY, inv(9)(p12q13) |
| 6 | 46,XY, 22pstk+ |
| 7 | 46,XY, t(15;17)(p10;q10) |
| 8 | 46,XY, t(4;7)(q21;q36) |
| 9 | 46,XY, 13pss |
| 10 | 46,XY, inv(18)(p11;q12) |
| 11 | 46,XY, inv(9)(p12q13) |
| 12 | 46,XY, t(14;15)(q22;q24) |
| 13 | 46,XY, t(2;19)(q13;q13.1) |
| 14 | 47, XYY |
| 15 | 46,XY, inv(9)(p12q13) |
| 16 | 46,XY, t(12;16)(q24.3;q12.1) |
| 17 | 45,XY, rob(15;21)(q10;q10) |
| 18 | 45,XY, rob (13;14) (q10;q10) |
| 19 | 46,XX, t(1;18)(p13.3;q21.1) |
| 20 | 46,XY, 15p+ |
| 21 | 45,XY, rob(13;14) (q10;q10) |
| 22 | 45,XX, rob (14;22) (q10;q10) |
| 23 | 46,XX, t(12;13)(q22;q34) |

PGD: pre-implantation genetic diagnosis
